# Mayaro virus in Latin America and the Caribbean

**DOI:** 10.26633/RPSP.2020.14

**Published:** 2020-02-11

**Authors:** Niloofar Ganjian, Ana Riviere-Cinnamond

**Affiliations:** 1 Milken Institute School of Public Health The George Washington University Washington, D.C United States of America Milken Institute School of Public Health, The George Washington University, Washington, D.C., United States of America.; 2 Pan American Health Organization Pan American Health Organization Washington, D.C United States of America Pan American Health Organization, Washington, D.C., United States of America.

**Keywords:** Disease outbreaks, arboviruses, infection, Latin America, West Indies, Brotes de enfermedades, arbovirus, infección, América Latina, Indias Occidentales, Surtos de doenças, arbovírus, infecção, América Latina, Índias Ocidentais

## Abstract

**Objectives.:**

To assess the distribution of Mayaro virus (MAYV) in Latin America and the Caribbean and evaluate existing country-level MAYV surveillance mechanisms.

**Methods.:**

Research was conducted from May 2018 through May 2019 to collect data from academic literature on Mayaro fever in Latin America and the Caribbean. PubMed, ClinicalKey, Scopus, Nature, SciELO, LILACS, and Google Scholar were searched for peer-reviewed journal articles, and data from health authorities, including the Pan American Health Organization (PAHO) and ministries of health, was also sought. MAYV-related publications published from 1954 through 2019 were screened. Publications that added to the overall understanding of MAYV, including its geographical and epidemiological distribution, were included in this report.

**Results.:**

A total of 901 MAYV cases have been reported in humans in countries in Latin America and the Caribbean. Since its discovery in 1954 in Trinidad and Tobago, MAYV has been isolated from individuals living in Argentina, Bolivia, Brazil, Ecuador, French Guiana, Haiti, Mexico, Panama, Peru, and Venezuela. Of those 901 cases, 42 of them were reported exclusively by health authorities. In contrast, 843 confirmed and presumptive autochthonous cases and an additional 16 imported cases were identified in academic literature. No country-level surveillance mechanisms for MAYV were recorded in academic literature or by health authorities.

**Conclusions.:**

This report demonstrates that MAYV surveillance efforts are limited in comparison to the virus’s presence in Latin America and the Caribbean, highlighting the importance of enhancing arboviral surveillance systems in the affected countries.

## RESUMEN

**Objetivos.:**

Evaluar la distribución del virus Mayaro (MAYV) en América Latina y el Caribe y los mecanismos de vigilancia de nivel nacional.

**Métodos.:**

Entre mayo de 2018 y mayo de 2019 se llevó a cabo una revisión bibliográfica sobre la fiebre de Mayaro en América Latina y el Caribe en las bases de datos PubMed, ClinicalKey, Scopus, Nature, SciELO, LILACS y Google Académico para identificar artículos en revistas arbitradas; también se revisó información suministrada por las autoridades sanitarias, entre ellas la Organización Panamericana de la Salud y los ministerios de salud. Se revisaron artículos relacionados con MAYV publicados entre 1954 y 2019. En este informe se incluyeron artículos que contribuyeran a la comprensión general sobre el MAYV, incluida su distribución geográfica y su epidemiología.

**Resultados.:**

Se han reportado 901 casos de MAYV en seres humanos en 11 países de América Latina y el Caribe. Desde su descubrimiento en 1954 en Trinidad y Tobago, MAYV ha sido aislado de personas en Argentina, Bolivia, Brasil, Ecuador, Guayana Francesa, Haití, México, Panamá, Perú y Venezuela. De estos 901 casos, 42 fueron reportados exclusivamente por las autoridades sanitarias. A la vez, en la bibliografía se identificaron 844 casos autóctonos confirmados o presuntivos y 15 casos importados. A través de la bibliografía o las autoridades sanitarias no se identificaron mecanismos de vigilancia de nivel nacional para el MAYV.

**Conclusiones.:**

En América Latina y el Caribe la vigilancia del MAYV es limitada en comparación con la presencia del virus en la región; es necesario mejorar los sistemas de vigilancia de arbovirus en los países afectados.

## RESUMO

**Objetivo.:**

Analisar a distribuição do vírus Mayaro (MAYV) na América Latina e Caribe e avaliar os mecanismos nacionais de vigilância.

**Métodos.:**

Dados da literatura acadêmica sobre a febre Mayaro na América Latina e Caribe foram coletados de maio de 2018 a maio de 2019. Foram pesquisadas as bases de dados PubMed, ClinicalKey, Scopus, Nature, SciELO, LILACS e Google Acadêmico para identificar artigos em revistas revisadas por pares; também foram examinados dados de autoridades sanitárias, incluindo a Organização Pan-Americana da Saúde (OPAS), e dos ministérios de saúde. Artigos relacionados com MAYV publicados de 1954 a 2019 foram revisados. Este relatório incluiu artigos que contribuíssem para o entendimento geral do MAYV, com informações sobre distribuição geográfica e epidemiologia.

**Resultados.:**

Foram registrados 901 casos de MAYV em humanos em 11 países da América Latina e do Caribe. Desde a sua descoberta em 1954 em Trinidad e Tobago, o MAYV foi isolado em indivíduos na Argentina, Bolívia, Brasil, Equador, Guiana Francesa, Haiti, México, Panamá, Peru e Venezuela. Desses 901 casos, 42 foram comunicados exclusivamente pelas autoridades sanitárias. Por sua vez, 844 casos autóctones confirmados ou suspeitos e 15 casos importados foram descritos na literatura. Nenhum mecanismo nacional de vigilância do MAYV foi identificado na literatura ou pelas autoridades sanitárias.

**Conclusões.:**

Na América Latina e Caribe, a vigilância do MAYV é limitada em contraste com a presença do vírus na região; é importante melhorar os sistemas de vigilância desse arbovírus nos países afetados.

Arboviral diseases share many common characteristics, making them difficult to discern from one another. However, due to changes in anthropogenic factors and viral mutations, arboviruses have the potential to emerge or reemerge throughout the Americas. In order to properly allocate resources and develop interventions to prevent the spread of arboviruses, it is important to make the diseases discernible from one another.

Mayaro virus (MAYV) is a positive-sense, enveloped, singlestranded RNA alphavirus classified in the Semliki Complex. MAYV is the etiological agent of Mayaro fever (1). The Semliki Complex is made up of eight viruses of importance in the medical and veterinary fields: Bebaru, Chikungunya, Getah, Mayaro, O’nyong-nyong, Ross River, Semliki Forest, and Una (1). Due to the fact that these eight viruses belong to the same serological complex, it is difficult to achieve an accurate diagnosis of the viruses outside of well-equipped laboratory settings (1). Further, the virus is closely related to and cocirculates with Venezuelan equine encephalitis and Chikungunya viruses (2).

MAYV is maintained in an enzootic cycle through continuous evolutions of transmission between diurnal mosquitoes from the forest canopy to nonhuman primates, rodents, marsupials, and birds (3, 4). Similar to the yellow fever virus life cycle, the transmission of MAYV to humans is believed to occur in the rural cycle, in which mosquitoes spread the virus to humans who live near to or make frequent use of forest habitats (4). To date, human cases of Mayaro fever have mostly occurred due to accidental spillover from the sylvatic cycle (3).

Transmission of MAYV to humans primarily occurs by the bite of female mosquitoes from the *Haemagogus* genus in the dipteran family Culicidae, which are not known to be anthropophilic (1, 4). In order to assess whether an urban cycle of MAYV could be initiated and sustained, laboratory experiments were conducted to assess the vector competence of anthropophilic mosquitoes (1, 5). Studies showed that MAYV can infect *Aedes albopictus,* but *Aedes aegypti* demonstrated limited opportunity for MAYV transmission to humans (1, 5); however, the vector competence of the species was demonstrated and suggests the potential for urban transmission (5). MAYV-infected *Ae. Aegypti* and *Culex quinquefasciatus* have also been found in the city of Cuiabá, in the state of Mato Grosso, Brazil (6). MAYV was first isolated from *Mansonia venezuelensis* collected from the Rio Grande Forest in Trinidad and Tobago (5). A small number of *Psorophora ferox/albipes*, *Ae.* (*Ochlerotatus) serratus*, *Sabethes* spp., *Culex* spp., and *Haemagogus janthinomys* have also contained isolates of the virus (5).

Further evidence of the virus’s potential to emerge in new areas was provided when sheep, caiman, and equids were sampled from 16 cattle ranches across the Pantanal region of Brazil from October 2009 through January 2011 (7). Results from the study showed that 10 of the 748 equids were seropositive for MAYV, demonstrating the first evidence of MAYV circulation in the southern Pantanal. Since equids tested negative for MAYV in previous investigations, the authors of the study suggested that MAYV circulation in the Pantanal might be a recent occurrence. More research is needed to identify amplifier hosts and vectors, and to understand the ecology of the transmission of the virus (7).

Clinical symptom of Mayaro fever tend to be nonspecific and include high-grade fever, debilitating arthralgia, headache, retro-orbital pain, myalgia, vomiting, diarrhea, and maculopapular rash (9). Similar to Chikungunya, MAYV infection can also lead to the development of long-term arthralgia (9). To date, no studies have shown whether individuals infected with MAYV can present as asymptomatic.

Due to the generic nature of the symptoms of alphaviruses, misdiagnosis can occur. Diagnosis of MAYV relies on clinical diagnosis, which may contribute to the misclassification of MAYV cases for dengue, Zika, Chikungunya, or other arboviruses (3, 4). The diagnostic procedures for MAYV include serological detection through the detection of antibodies in the host serum; molecular detection through reverse transcription polymerase chain reaction (RT-PCR); and viral isolation in a cell culture (4). The reference standard for MAYV diagnosis is viral isolation (4). Enzyme-immune assays, hemagglutination inhibition, immunofluorescence, or neutralization methods are useful serological methods for the detection of antibodies (4). Diagnostic misdiagnoses can occur among viruses of the *Alphavirus* genus due to cross-reactivity among viruses with common antigenic sites (1). Further, the short viremic period of MAYV may make it difficult to accurately isolate the virus from blood (4). As a result, the exact number of MAYV cases is difficult to determine in both clinical and serological detection outside of well-equipped laboratories (1).

Disease prevention relies on vector control and personal protection measures (10). Case management of MAYV relies solely on the treatment of symptoms with analgesics. Efforts to develop other control methods have been recorded. For example, the antiviral effects of thienopyridine derivatives have been evaluated and found to affect the late early and late stages of MAYV replication (11). One study demonstrated that these substances could potentially act as a new class of antiviral drugs due to their bioavailability and low cytotoxicity (11). A live-attenuated vaccine against MAYV has also been developed. The vaccine was found to protect against lethal challenge in murine models, was highly immunogenic, and noninfectious to mosquitoes, but still required further preclinical development (12). A synthetic DNA envelope vaccine has also been evaluated in mice (13). T cell immunity and antibody immune responses were induced by the vaccine, and mice challenged with live MAYV after receiving the vaccine were protected against the disease (13). Despite these developments, licensed vaccinations and antiretroviral therapy are not currently available for MAYV (1, 4).

The objectives of this report were to assess the presence of MAYV in Latin America and the Caribbean while simultaneously evaluating country-level MAYV surveillance efforts in Latin American and Caribbean countries.

## MATERIALS AND METHODS

In order to assess the presence of Mayaro fever in Latin America and the Caribbean, databases that included PubMed, ClinicalKey, Scopus, Nature, SciELO, LILACS, and Google Scholar were searched periodically from May 2018 through May 2019. Materials in French, Spanish, Portuguese, and English were reviewed. The terms used and adapted to each database were “Mayaro fever,” “Mayaro virus,” “MAYV,” “Mayaro,” “Mayaro AND alphavirus,” “Mayaro AND Semliki Complex,” and “Mayaro AND epidemiology.” In PubMed, 90 articles were identified for the search term “Mayaro fever,” 221 articles for “Mayaro virus,” 79 articles for “MAYV,” 227 articles for “Mayaro,” 158 articles for “Mayaro AND alphavirus,” 9 articles for “Mayaro AND Semliki Complex,” and 71 articles for “Mayaro AND epidemiology.” (The other databases did not generate new, unique journal-article hits that were not in PubMed. Therefore, from this point forward, this report will discuss the journal-article hits only from PubMed.)

In addition, health authorities were consulted to obtain outbreak data. Also, the archives of the Pan American Health Organization were examined, and epidemiological bulletins were investigated on the ministry of health websites of countries with a reported presence of Mayaro (Argentina, Bolivia, Brazil, Ecuador, French Guiana, Haiti, Mexico, Panama, Peru, Suriname, Trinidad and Tobago, and Venezuela). Lastly, references listed in academic articles and health authority reports were manually examined to further identify MAYV data for this report.

We included in our analysis journal articles and other publications that were published between 1954 and 2019 and that provided a historical overview of Mayaro, hosts, transmission mechanisms of the virus, clinical manifestation, geographical distribution and epidemiology of the disease, diagnostic methods, case management, surveillance efforts, known risk factors, and prevention and control techniques. Studies were excluded from the report if they were unable to add to the overall understanding of MAYV in Latin America and the Caribbean, including studies that were too broad or too heavily focused on the microbiological and virological components of MAYV or did not contribute data to the analysis.

## RESULTS

**FIGURE 1.**
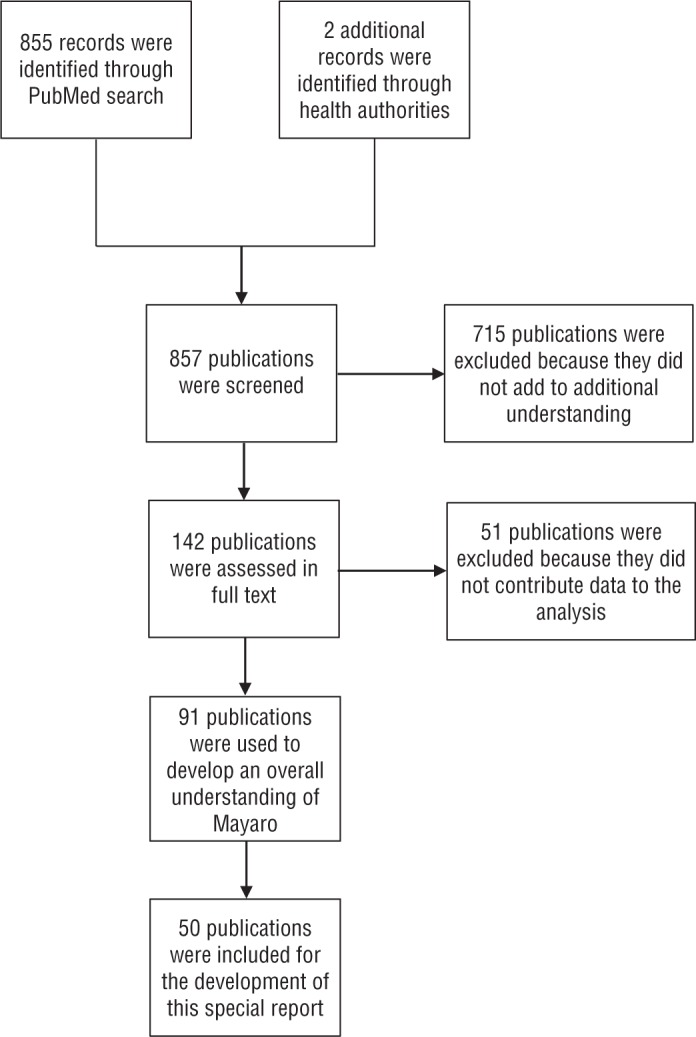
Study flow diagram, including the PubMed searching, done for the special report on Mayaro in Latin America and the Caribbean

***Source:*** This figure was prepared by the authors, based on the study results.

Periodic searches were carried out between May 2018 and May 2019 to assess the distribution of Mayaro fever in Latin America and the Caribbean. The study flow diagram, including with the PubMed searching, is given in Figure 1.

In this special report, 50 publications were included in the analysis and 41 were excluded. Of those 50 included publications, 49 of them were journal articles and the other was a health authority alert issued by the Pan American Health Organization (PAHO) in May of 2019.

**FIGURE 2.**
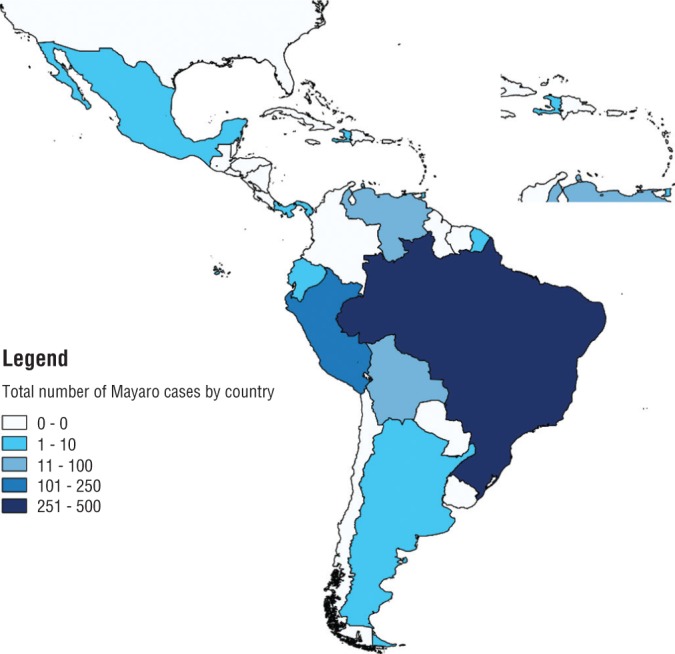
Total number of cumulative confirmed and presumptive Mayaro cases reported in academic literature and by health authorities, from Mayaro’s discovery in 1954 through May of 2019, by country^a^

***Source:*** This figure was prepared by the authors, based on the study results.

a The image does not include the 16 imported cases of Mayaro.

**TABLE 1.** Total number of cumulative confirmed and presumptive Mayaro cases reported in academic literature and by health authorities, from its discovery in 1954 through May 2019, by country^a^

Country	Number of Mayaro cases
Argentina	3
Bolivia	60
Brazil	495
Ecuador	6
French Guiana	1
Haiti	1
Mexico	2
Panama	1
Peru	230
Trinidad and Tobago	5
Venezuela	81

***Source:*** This table was prepared by the authors, based on the study results.

a The table does not include 16 imported cases of Mayaro.

As shown in Figure 2 and Table 1, MAYV cases have been found in Latin America and the Caribbean, with the highest number of cases occurring in Brazil and Peru. Of the 901 reported cases of MAYV, approximately 843 confirmed and presumptive autochthonous cases and 16 imported cases were identified in academic literature, and the remaining 42 cases were reported exclusively by a health authority (1, 4, 8, 14-37). Epidemiological bulletins from the ministries of health in Argentina, Bolivia, Brazil, Ecuador, French Guiana, Haiti, Mexico, Panama, Peru, Suriname, Trinidad and Tobago, and Venezuela made no mention of the presence of Mayaro.

Table 2 includes a descriptive overview of reported confirmed and presumptive cases of Mayaro, as well as the publications in which these cases were identified. The table provides information on the number of cases reported in an article, the lead author of the article in which the case(s) were reported, the country the case(s) originated in, the year the case(s) occurred, and the diagnostic procedure used to confirm the case(s).

**TABLE 2.** Epidemiological database of confirmed and presumptive Mayaro cases reported in academic literature and by health authorities, from Mayoro’s discovery in 1954 through May 2019, by country^a^

Reference source(s)	Country	Year cases were reported	No. of reported cases	Diagnostic procedures implemented to detect confirmed or presumptive MAYV cases
Diaz, 2003 (35)	Argentina	2001	3	Human sera of citizens aged 44-89 from Córdoba were collected and assessed at the Instituto Nacional de Previsión Social (PAMI) in 2001. 79 samples were tested by neutralization assay. Three out of the 79 sera were positive for Una virus (UNAV), a subtype of Mayaro. Due to the low titers of neutralizing antibodies detected, the UNAV infections could have occurred years ago but they demonstrate circulation of the virus in humans
Schmidt, 1959 (15)	Bolivia	1955	2	Uruma virus was isolated from the blood of 2 febrile Okinawan colonists living in the rainforest of Eastern Bolivia. The Uruma virus is now considered to be a strain of the Mayaro virus. Virus dilution neutralization tests were conducted on the sera of 21 healthy individuals that had not been involved in the outbreak, living in Puerto Cespedes. Acute and convalescent phase sera from the patient of whom the virus was first isolated, as well as convalescent phase sera from 38 Okinawan patients from whom acute phase sera was not obtained. Since no acute phase serum was obtained from the 38 Okinawans who experienced febrile illness, a definitive etiologic diagnosis was not established. 8 of the 21 healthy individuals living in Puerto Cespedes showed detectable antibodies to the virus. This demonstrates that infection with Uruma virus, or an antigenic relative, is common among residents of the rainforest.
Auguste, 2015 (14)	Bolivia	2007	12	Further information was not provided.
Forshey, 2010 (16)	Bolivia	2000-2007	6	3 confirmed cases (virus isolation, RT-PCR, or IgM seroconversion), and 3 presumptive cases (elevated IgM without 4-fold rise between acute and convalescent phases) were identified.
Forshey, 2010 (16)	Bolivia	2000-2007	3	1 confirmed case (virus isolation, RT-PCR, or IgM seroconversion), and 2 presumptive cases (elevated IgM without 4-fold rise between acute and convalescent phases) were identified.
Forshey, 2010 (16)	Bolivia	2000-2007	16	10 confirmed cases (virus isolation, RT-PCR, or IgM seroconversion), and 6 presumptive cases (elevated IgM without 4-fold rise between acute and convalescent phases) were identified.
Forshey, 2010 (16)	Bolivia	2000-2007	21	10 confirmed cases (virus isolation, RT-PCR, or IgM seroconversion), and 11 presumptive cases (elevated IgM without 4-fold rise between acute and convalescent phases) were identified.
Alves Esposito, 2017 (1)	Brazil	1955	6	On April 15, 53 of the 115 individuals that arrived at the lab with an illness had a fever. Only 34 of the 53 sera were inoculated and the remaining 18 were taken to the lab the following day. Four strains of the virus were isolated in this initial round of testing. A second round of testing was conducted on May 7. Samples were collected from the 37 people who were bled on April 15 and serum was collected from an additional 37 individuals that had not been bled previously. An additional two samples, from the sera samples of those who had not been previously bled, yielded virus strains.
Alves Esposito, 2017 (1)	Brazil	1977-1978	55	A total of 807 clinically suspected cases were recorded from a rubber plantation located in Belterra. This epidemic coincided with a yellow fever outbreak. 72 persons were examined, the virus being confirmed in 55 during the acute and subacute stages of the illness. 43 cases were detected with virus isolation and serology and 12 with serological conversion using HI tests.
Alves Esposito, 2017 (1); Munoz, 2012 (19)	Brazil	1978-1981	Number of cases not provided	Further information was not provided.
Alves Esposito, 2017 (1); Munoz, 2012 (19)	Brazil	1981-1991 (1981, 1984, 1988, 1991)	Number of cases not provided	Further information was not provided.
Alves Esposito, 2017 (1)	Brazil	1991	Number of cases not provided	Further information was not provided.
Pinto De Figueiredo, 2004 (34)	Brazil	1998-1999	8	8 557 serum samples were taken from March 1998-December 1999. Exanthematic diseases were tested from 1 107 dengue negative samples. Of those samples, 22 were tested for Mayaro. 8 of the 22 samples tested positive for Mayaro. Diagnoses were performed using serological tests to detect IgM antibodies by ELISA CDC/PAHO or with commercial kits.
Coimbra, 2007 (32)	Brazil	2000	3	Serum samples were collected four days and two months after the onset of symptoms. Virus isolation was obtained from only one acute blood sample, but convalescent sera samples revealed monotypic seroconversion to MAYV, and a final identification of MAYV was further obtained in a RT-PCR assay. The three men were visiting from other areas of Brazil to fish. The patients were infected between March 11-19, 2000
Terzian, 2015 (18)	Brazil	2000	1	MAYV was isolated from the patient, who had been fishing. Further information was not provided.
Terzian, 2015 (18)	Brazil	2004	1	A 27-year-old female patient was identified during an ongoing epidemiological survey in the area. The patient was malaria-negative and presented with fever and chills. There were no signs or symptoms of joint pain or swelling, but no follow-up was performed to confirm the presence or absence of arthralgia following the initial examination of the patient. The patient’s RT-PCR sample was positive for MAYV. Bayesian phylogeny supported that the strain of MAYV isolated in this case was closely related to the Bolivian strains isolated between 2002 and 2006, a finding not completely unexpected given the proximity of the state of Acre to Bolivia.
Alves Esposito, 2017 (1); Azevedo, 2009 (9)	Brazil	2008	36	A dengue-like illness was reported in the Pau d’Árco settlement, a rural community in the middle of a native forest. 105 individuals were examined in house-to-house surveys. 53 lived in the settlement and were agricultural workers, and 52 were agronomy students at the public university in the city of Belém, Pará. IgM was detected in 36 (34%) serum samples. Of those 36 samples, 23 (64%) were collected from residents of the settlement, and 13 (36%) were from residents of the city of Belém, Pará, and the city of Ananindeua, Pará; these persons had visited the settlement area for a week. 36 of these individuals had IgM antibodies against MAYV.
Munoz, 2012 (19)	Brazil	2011	33	Further information was not provided.
Pinheiro, 1977 (20)	Brazil	1973-1974	13	Studies were carried out in patients who attended the hospital in the city of Altamira and the health posts. Most of these patients were colonists. Between January 1973 and June 1974, 549 blood samples were taken. Only one arbovirus strain of Mayaro was isolated from a febrile case. The low rate of virus isolation is likely due to the fact that the majority of patients were bled after the third day of illness, when viremia tends to be absent. 160 of 832 demonstrated serological conversion to arboviruses using HI test from Jan 1973-June 1974. 60 of the seroconversions where to arboviruses of other antigenic groups; 12 were for MAYV
Tavares-Neto, 2004 (21)	Brazil	1999-2000	2	Tests were done on individuals in August 1999 and again in January 2000. Results reflect seroconversion during that period.
Alves Esposito, 2017 (1)	Brazil	2007-2008	33	IgM antibodies against MAYV reported in 33 individuals. A viral genome was detected for one of the cases in the city of Manaus. Further information was not provided.
Cruz, 2009 (22)	Brazil	2007-2008	5	The 113 and 102 serum samples that presented titers equal to or greater than 40 by HI test to Oropouche virus and MAYV, respectively, were analyzed by ELISA for detection of IgM antibodies, and identified 23 recent infections for Oropouche viruses and 5 for MAYV.
Alves Esposito, 2017 (1); Zuchi, 2014 (23)	Brazil	2011-2012	15	15 out of 604 patients during a dengue outbreak tested positive for MAYV RNA
Vieira, 2015 (46)	Brazil	2011-2012	6	The presence of Brazilian arboviruses was investigated in the sera of 200 patients with acute febrile illness during a dengue outbreak in the city of Sinop. Results showed that 6 samples were positive to MAYV. Molecular and evolutionary analyses also showed that two MAYV genotypes are co-circulating in Mato Grosso. DENV negative samples were tested by using multiplex-nested-PCR to identify MAYV. If it had not been for the intensive RT-PCR surveillance for the other arboviruses, the detection of MAYV cases might have been missed.
Alves Esposito, 2017 (1)	Brazil	2014-2016	183	Further information was not provided.
Brunini, 2017 (48)	Brazil	2014-2015 (June)	16	Of 27 CHIKV-negatives samples, 16 of the samples tested by HI were reactive to alphaviruses. 15 of the samples were confirmed positive for MAYV by IgM MAC-ELISA and one sample was borderline. The article did not mention whether the 16 samples had a negative RT-PCR MAYV result in the acute phase specimen or whether an acute phase specimen was taken. Since this information was not provided, it cannot be inferred that the IgM MAC-ELISA results are confirmed or probable cases. All the individuals that tested positive for MAYV IgM had travelled to rural areas in the 15 days before the onset of symptoms. Identification of recent infections in rural and forested areas around the city of Goiânia suggests active foci in the forest cycle where a MAYV forest cycle is established.
Nunes, 2009 (24)	Brazil	Not specified (article published in 2009)	78	Among the samples, 78 monotypic reactions were observed for MAYV. Of those, 10 presented anti-MAYV IgM antibodies. Of those 10 samples, 4 were from the municipality of Trairão and 6 came from the municipality of Novo Progresso.
Estofolete, 2016 (47)	Brazil	Not specified (article published in 2016)	1	A case report revealed that an HIV-infected patient that returned from a work trip to the Amazon basin presented symptoms of febrile illness. After testing negative for DENV and CHIKV, MAYV was confirmed by RT-PCR and subsequently followed by next-generation sequencing and phylogenetic reconstruction. The test was developed in the in-house laboratory for the E1 gene. The interplay between arboviral diseases and HIV is poorly understood. In theory, HIV-induced immunosuppression can lead to more atypical and aggressive manifestations of arboviral infections.
Forshey, 2010 (16)	Ecuador	2000-2007	1	1 confirmed case (virus isolation, RT-PCR, or IgM seroconversion) was identified.
PAHO, 2019 (38)	Ecuador	2019	5	34 samples that were negative for Zika, leptospirosis, dengue, and Chikungunya were tested for Mayaro. Five of the samples were positive. Two of the cases were from the city of Guayaquil, one was from the city of Portoviejo, one case was from the city of Santo Domingo, and one other case was from the city of Babahoyo. The cases were detected through laboratory surveillance of MAYV.
Talarmin, 1998 (25)	French Guiana	1996	1	Mayaro virus was isolated from the acute-phase serum of a woman who had been living in French Guiana for several months and presented with a febrile illness for two days. Immunofluorescent antibody testing with specific mouse antibody was initially performed and confirmed by plaque-reduction neutralization and RT-PCR. A total of 1,962 sera (from 896 women and 1066 men) were tested for antibodies to MAYV. Antibodies to this virus were found in 124 (6.3%) sera but IgM was only detected once.
Lednicky, 2016 (26)	Haiti	2015	1	The patient was an eight-year-old boy from a rural/semi-rural area of Haiti, reflecting an ecologic setting that differs greatly from forest Amazon regions where many of the other reported MAYV infections have occurred. Little is known about vectors for MAYV in Haiti. A blood sample was collected, and RNA was extracted via RT-PCR. The sample confirmed DENV-1. In addition, an alphavirus amplicon similar in size to the expected size of MAYV was also detected in the viral RNA extracted from the infected Vero cells. Sequencing of the amplicon confirmed that it corresponded to MAYV.
Navarrete-Espinosa, 2006 (27)	Mexico	2001	2	35 patients at the Mexican Institute of Social Security were isolated and tested by PCR and two tested positive for MAYV. These patients were hemorrhagic cases; one patient died.
Srihongse, 1973 (33)	Panama	1966-1967	1	A significant rise of antibody titers was identified in the sera of an individual who was positive for MAYV. The patient was part of a group of U.S. citizens who were conducting sea-level-canal feasibility studies in Panama and Colombia. Due to study group crossover, it was difficult to ascertain in which country the virus was acquired.
Tesh, 1999 (28)	Peru	1995-1998	26	From April 1995 to April 1998, 26 cases of MAYV were diagnosed in people living and working in Peru. 20 of the cases were isolated from acute-phase serum samples (confirmed cases), and the remaining 6 cases (presumptive) were diagnosed by serology. Demographic data was only collected on 24 individuals (13 females, 11 males). Ages of the 24 patients ranged from 9-65 years. 17 patients had occupational information available, including general laborer, agricultural worker, missionary, biologist, soldier, student and housekeeper. It was not clear which of the cases were presumptive or confirmed at each location. 15 of the 26 cases were detected accidentally by the isolation of MAYV from serum samples from patients with presumptive yellow fever or dengue, and 11 of the 26 cases were detected during prospective surveillance of patients with febrile illness in Iquitos.
Institutional study, Peruvian Ministry of Health, 2005 (36)	Peru	2000-2001	1	Between May 2000 and July 2001, a longitudinal and descriptive study was performed across four health facilities. Febrile patients aged 5-65 years with negative smears of Bartonella and malaria were included. IgM-IgG was performed for Mayaro; MAYV IgM was found in one patient.
Forshey, 2010 (16)	Peru	2000-2007	5	4 confirmed cases (virus isolation, RT-PCR, or IgM seroconversion), and 1 presumptive case (elevated IgM without 4-fold rise between acute and convalescent phases) were identified.
Forshey, 2010 (16)	Peru	2000-2007	2	2 presumptive cases (elevated IgM without 4-fold rise between acute and convalescent phases) were identified.
Forshey, 2010 (16)	Peru	2000-2007	20	10 confirmed cases (virus isolation, RT-PCR, or IgM seroconversion), and 10 presumptive cases (elevated IgM without 4-fold rise between acute and convalescent phases) were identified.
Forshey, 2010 (16)	Peru	2000-2007	107	48 confirmed cases (virus isolation, RT-PCR, or IgM seroconversion), and 59 presumptive cases (elevated IgM without 4-fold rise between acute and convalescent phases) were identified.
Forshey, 2010 (16)	Peru	2000-2007	16	11 confirmed cases (virus isolation, RT-PCR, or IgM seroconversion), and 5 presumptive cases (elevated IgM without 4-fold rise between acute and convalescent phases) were identified.
Halsey, 2013 (29)	Peru	2010-2013	16	16 of the 2 094 febrile participants enrolled had Mayaro fever. Of the 16 persons with Mayaro fever, 11 had MAYV isolated by cell culture assays (11 in both Vero 76 and C6/36), 13 were MAYV positive by RT-PCR, and all had IgM ELISA seroconversion between the acute phase and 20-day follow-up visits. In all 16 participants, no IgM ELISA seroconversion occurred for endemic non-alphavirus viruses. Four participants showed IgM ELISA seroconversion against another alphavirus, Venezuelan equine encephalitis virus, but these 4 all had MAYV identified by immunofluorescence assay and by RT-PCR.
PAHO, 2019 (38)	Peru	2018	35	Additional information was not provided.
PAHO, 2019 (38)	Peru	2019	2	One case originated from Quispicanchis, Cusco Region and the other case originated
Anderson, 1957 (37)	Trinidad and Tobago	1954	5	from La Mar, Ayacucho Region. The virus was isolated from the blood samples of four male forest workers and one female urban dweller who presented with febrile illness.
Torres, 2004 (30)	Venezuela	2000	4	Serum samples were tested using IgM MAC-ELISA. Assay of serum samples were obtained 3 months after onset of symptoms and again three months after the initial samples were taken. Three of the family members showed high specific Mayaro viral IgM antibody ranging from 3 200 to 6 400 and IgG antibody titers ranging from 6 400 to 12 800. Testing of samples from the fourth patient were positive for MAYV IgG antibody only. MAYV may have been circulating in a cycle involving Haemagogus mosquitoes and red howler monkeys.
Auguste, 2015 (14)	Venezuela	2010	77	77 cases were reported and 19 were confirmed as seropositive. Of 19 acute-phase serum samples, MAYV was isolated from 6 symptomatic patients. 27 complete genomes were sequenced, which facilitated the detection of a new genotype, referred to as N. The Mayaro fever outbreak at La Estación in 2010 is noteworthy because, with the exception of a family found to be seropositive, it represents the first outbreak documented in Venezuela.

***Source:*** This table was prepared by the authors, based on the study results.

a This table does not include the 16 imported cases of Mayaro.

CHIKV, Chikungunya virus; DENV, dengue virus; HI, hemagglutination inhibition; IgM MAC-ELISA, IgM antibody capture enzyme-linked immunosorbent assay; MAYV, Mayaro virus; PAHO, Pan American Health Organization; RT-PCR, reverse transcription polymerase chain reaction.

The lack of standardization in Mayaro case definitions and laboratory diagnostics weaken the robustness of reported case data; however, the data was incorporated in this report due to the limited availability of MAYV case data to create the first epidemiological database for Mayaro. To date, epidemiological data on Mayaro continues to be descriptive in nature. Given the lack of accurate and consistent case information, incidence rate calculations were not attempted. Rather, an overview of the number of reported cases and/or epidemics of MAYV was given. Data on MAYV mortality was extremely limited. Of the 901 reported MAYV cases, only one fatality was recorded in academic literature (27). Lastly, MAYV attack rates were not available in the existing literature.

### Argentina

Three cases of Una virus (UNAV), a subtype of Mayaro, were discovered in the sera of three individuals from central Argentina in 2001 (35). Due to the low titers of neutralizing antibodies detected, it was difficult to ascertain when the individuals became infected with UNAV; nonetheless, the circulation of UNAV in Argentina was demonstrated (35).

### Bolivia

A total of 60 cases were identified from 1955 through 2007 in Bolivia. In the first instance, Uruma virus, now considered to be a strain of Mayaro, was isolated from the blood of two febrile Okinawan colonists in 1955 (15). Another article reported that 12 cases of MAYV were found in 2007, but no further information was provided about the cases (14). The remaining 46 MAYV cases in Bolivia were identified from 2000 to 2007 in a study done in three departments of the country (16).

### Brazil

The country with the highest number of recorded MAYV cases in the Americas is Brazil. Of a total of 495 cases, 199 of them were identified in the state of Goiás, and 194 took place in the state of Pará. Cases were also identified in the states of Acre, Amazonas, and Mato Grosso and the city of São Paulo. The virus is considered to be endemic in the northern, western, and central portions of the country (1).

### Ecuador

The first case of MAYV in Ecuador was identified during a study that took place from 2000 to 2007 (16). The patient lived in the city of Guayaquil, in the province of Guayas (16). In May of 2019 an epidemiological bulletin released by PAHO reported that an additional five cases of Mayaro were detected in Ecuador (38). These cases were identified in samples that tested negative for leptospirosis, dengue, Chikungunya, and Zika (38) and that had originated from individuals in the cities of Guayaquil, Babahoyo, Portoviejo, and Santo Domingo (38).

### French Guiana

The first case of MAYV in French Guiana was identified in 1996 (25). The patient was a female who had been living in French Guiana for several months and had presented with symptoms for two days (25).

### Haiti

Only one case of MAYV has been recorded in Haiti. In 2015, an eight-year-old boy from a rural/semirural area of the Ouest department presented febrile symptoms and tested positive for MAYV (26). This case demonstrates an instance where the ecological settings that precipitated the emergence of this case differed from the sylvan Amazon regions where MAYV infections have generally occurred (26).

### Mexico

A total of two cases of MAYV have been found in Mexico. These cases were identified in 2001, when 35 patients at the Mexican Institute of Social Security in the state of Veracruz underwent testing for MAYV (27). Two of the patients tested positive for the virus and displayed symptoms characteristic of hemorrhagic cases (27). Of the two cases, one patient died (27). This has been the only recorded case of fatality due to MAYV (27).

### Panama

One case of MAYV was detected in a study carried out from 1966 through 1967 (33). In this instance, a citizen of the United States of America who was conducting studies assessing the feasibility of routes for a new, sea-level canal in Panama or Colombia demonstrated a significant rise in viral MAYV antibodies (33). Unfortunately, due to study group cross-over, it was unclear whether the virus was acquired in Panama or Colombia (33).

### Peru

Peru had the second-largest number of reported cases. A total of 230 cases were identified, with the highest number reported in the region of Loreto. Mayaro was also recorded in the regions of Madre de Dios, Junín, Cusco, Huánuco, Ucayali, San Martín, Tumbes, and Ayacucho.

### Trinidad and Tobago

Trinidad and Tobago was the location where MAYV was first isolated, from blood samples from one urban-dwelling female and four male forest workers (1, 37). Since the virus was first discovered in the county of Mayaro, it was subsequently named for that area.

### Venezuela

There have only been two recorded instances of MAYV infection in Venezuela. In 2000, MAYV was identified in a family of four in the Barlovento subregion of the state of Miranda (30). The second instance of MAYV was in the village of La Estación, where 77 cases were reported, of which 19 of them were confirmed as being seropositive (14).

### Imported cases

A total of 15 imported cases of Mayaro have been reported in the academic literature (9, 28, 39-45). These cases impacted individuals from Canada, France, Germany, the Netherlands, Switzerland, and unidentified parts of North America. The patients had acquired MAYV during travel in Bolivia, Brazil, Ecuador, French Guiana, Peru, and Suriname (9, 28, 39-45).

### Mayaro virus surveillance efforts

To date, no standardized case definitions or laboratory diagnosis algorithms have been recorded for Mayaro fever at the regional or country levels in Latin America and the Caribbean. Similarly, information on country-level MAYV surveillance efforts in Latin America and the Caribbean were not available in academic literature or from health authorities. However, many of the cases reported in academic literature came about from the differential diagnosis of sera that were negative for other, more well-known arboviral diseases (1, 18, 21-23, 26, 34, 39, 42, 46-48).

## DISCUSSION

MAYV was first isolated in 1954 in Trinidad and Tobago and has since been reported in Central and South America, circulating through high-density tropical forests (1, 4, 8, 14-37). A total of 901 MAYV cases have been reported in 10 Latin American countries and 1 Caribbean country. Of these 901 cases, 843 autochthonous and 16 imported cases were reported in academic literature, and the remaining 42 cases were exclusively reported by health authorities (1, 4, 8, 14-37). Given the extent of arboviral diseases in Latin America and the Caribbean, MAYV may pose a similar public health threat in the area.

As with other arboviral diseases, such as yellow fever, that have an urban cycle through *Ae. aegypti*, the MAYV case in Haiti highlights the need for vector competency research and demonstrates the potential for the MAYV transmission cycle to shift into an urban cycle. Urbanization of MAYV could establish new outbreaks of greater number throughout Latin America and the Caribbean and result in a wider distribution of MAYV, including circulation in North America and Europe (9, 26). Changes in the natural environment and the resulting impacts on interactions between host and vector populations could also expand the geographic distribution of MAYV in Latin America and the Caribbean (49). These changes could include the globalization of human and animal transportation, climate change, deforestation, human colonization, urbanization, mining, and infrastructure construction (dams and highways) (10, 28, 49). Increased air travel and globalization is expected to boost the number of imported cases of MAYV (9, 44). As such, MAYV demonstrates the potential to emerge in new areas and reemerge in existing areas and become a significant public health issue in the Western Hemisphere (10). Findings from our research coincide with previous research.

Cases of MAYV may be going undetected due to a lack of awareness among the medical community. For example, since diagnostic testing is not widely available and little is known of MAYV outside of endemic areas, imported cases may be getting clinically misdiagnosed as dengue. Within Latin America and the Caribbean, coinfection with other arboviral diseases may also misguide the diagnosis of MAYV, further demonstrating that the number of reported cases of MAYV only represent a portion of those that have occurred (9). It is also important to note that many of the cases of MAYV that were identified in academic literature were only detected because of extensive testing for arboviral viruses such as dengue and Chikungunya, further pointing to the need for differential testing for arboviral diseases. Lastly, since clinical symptoms of MAYV are generic, treatment may not be sought after and cases could go undetected. This would present a large obstacle to timely outbreak detection.

Data constraints must be considered when evaluating the outcomes of this research. The first limitation to this research includes the potential overestimation of the total number of presumptive MAYV cases found in academic literature due to serological cross-reaction between alphaviruses. Cross-reaction could also be a concern since diagnostic techniques were not uniform throughout the studies we included. Finally, many cases of MAYV may be unaccounted for both due to similarities between the clinical symptoms of MAYV and other arboviral diseases and coinfection.

Findings from this research demonstrate that MAYV surveillance efforts are limited in comparison to the virus’s epidemic potential, highlighting the importance of enhancing arboviral surveillance systems to include the differential diagnosis of MAYV. Given that the number of known cases of MAYV were primarily drawn from academic literature, country-level surveillance systems need to be strengthened to improve the data collection capabilities of health authorities.

Specific recommendations for MAYV surveillance include standardized lab capacities, the proactive seeking of cases, and the creation of standardized case definitions and laboratory diagnosis algorithms. While these recommendations are generally applicable to other arboviral diseases, the application of these recommendations specifically for MAYV will allow epidemics to be identified before they become problematic and also help the data collected to serve as a bridge between research and public health practice. Capacities and differential diagnosis algorithms need to be strengthened to ensure that labs are able to accurately distinguish among arboviral viruses. It is also important that labs be considered an integral data source for surveillance systems. Other recommendations include strengthening entomological surveillance and creating a global arboviral database. Public health practitioners and clinicians should also be made aware of the other arboviral diseases that exist, to be better able to discern the differences between them. Lastly, increased research interest will allow for the ongoing exploration of potentially epidemic diseases, such as Mayaro fever.

### Author contributions.

Dr. Ana Riviere-Cinnamond conceived the original idea for the special report, and Niloofar Ganjian collected the data, analyzed the data, interpreted the results, and wrote the paper. Both authors reviewed and approved the final version.

### Acknowledgments.

The publication would not have been possible without the intellectual contributions and expert advice provided throughout the process of compiling this special report. The authors would especially like to thank Dr. Sylvain Aldighieri, Dr. Enrique Perez-Gutierrez, Dr. Jairo Andres Mendez Rico, Dr. Juliana Leite, Jisoo Kim, Tshewang Choden Dorji, and Alexandra Gomes.

### Funding.

None declared.

### Disclaimer.

The authors hold sole responsibility for the views expressed in the manuscript, which may not necessarily reflect the opinion or policy of the *RPSP/PAJPH* and/or PAHO.
